# Characteristics and surgical outcomes of cleft palate in kabuki syndrome: a case series of 11 patients

**DOI:** 10.1186/s12887-021-02852-4

**Published:** 2021-09-03

**Authors:** Jong-Ho Kim, Jiwon Kang, Joon Seok Oh, Taeseon Ahn, Baek-kyu Kim, Rong-Min Baek

**Affiliations:** Department of Plastic and Reconstructive Surgery, Seoul National University College of Medicine, Seoul National University Bundang Hospital, 82 Gumi-ro 173beon-gil, Bundang-gu, Seongnam, 463-707 South Korea

**Keywords:** Kabuki syndrome, Cleft palate, Pharyngeal flap surgery

## Abstract

**Objective:**

A significant number of patients with KS have cleft palate (CP) or submucous cleft palate (SMCP) and show delayed speech development. However, few reports have discussed the characteristics of CP in KS and the outcomes of postoperative speech development. The purpose of this study was to investigate the characteristics and surgical outcomes of CP in patients with KS, and to discuss the importance of the diagnosis of CP or SMCP.

**Methods:**

We conducted a retrospective study on patients with clinically diagnosed KS who underwent palatoplasty. Clinical and surgical data were collected from patients’ medical records, and velopharyngeal function was evaluated using nasopharyngoscopy and speech analysis.

**Results:**

In 11 cases, 5 patients had CP (45.5%) and 6 had SMCP (54.5%). Four patients who were genetically tested had a pathogenic variant of *KMT2D*. Seven of nine patients (77.8%) who underwent conventional palatoplasty showed velopharyngeal insufficiency and hypernasality. All patients who underwent pharyngeal flap surgery achieved velopharyngeal competency. Statistical analysis revealed a statistically significant difference in postoperative results between non-syndromic and KS patients.

**Conclusion:**

Patients with SMCP may be more common than previously reported. The results showed that it is difficult to produce optimal results with conventional palatoplasty; therefore, pharyngeal flap surgery should be considered as a treatment to obtain favorable results. Pharyngeal flap surgery in patients with KS should be carefully designed based on speech evaluation and nasopharyngoscopic findings.

**Supplementary Information:**

The online version contains supplementary material available at 10.1186/s12887-021-02852-4.

## Introduction

Kabuki syndrome (KS [MIM: 147920 and 300,867]), first reported by Niikawa et al., is a syndrome of multiple congenital anomalies [1]. Niikawa et al. suggested the term “Kabuki make-up syndrome” due to the characteristic facial features resembling the make-up worn in the traditional Japanese play “Kabuki”. A clinical diagnosis based on unique facial features, as reported by Niikawa and Kuroki, is the most commonly used diagnostic tool [2]. The facial features of patients with KS include long palpebral fissures with slight ectropion of the lateral third of the lower eyelid and sparse lateral eyebrows [3]. In addition, patients with KS show a variable range of abnormalities including mild mental retardation, hearing difficulty, cardiac anomaly, and skeletal instability. In 2010 and 2012, pathogenic variants of lysine-specific methyltransferase 2D (*KMT2D*) and lysine-specific demethylase 6A (*KDM6A*) were reported to cause KS [4, 5]. As gene mutations became a critical diagnostic tool, new diagnostic criteria were suggested [6]. Based on these criteria, the authors suggested two major diagnostic criteria: pathogenic variants in *KMT2D* or *KDM6A* and typical dysmorphic features. Typical dysmorphic features include long palpebral fissures with eversion of the lateral third of the lower eyelid and two or more of the following: (1) arched and broad eyebrows with the lateral third displaying notching or sparseness; (2) short columella with depressed nasal tip; (3) large, prominent or cupped ears; and (4) persistent fingertip pads. Heterozygous mutations in *KMT2D* have been identified in approximately 60–70% of Patients with KS and mutations in *KDM6A* account for 5–8% of patients with KS [7]. Both genes encode for proteins that affect the epigenetic regulation of transcriptionally active chromatin by interacting with each other in the protein complex [8]. A significant number of patients with KS have cleft palate (CP) or submucous cleft palate (SMCP) and show delayed speech development [9]. Although the etiologic roles of both mutations have been proposed in the function of several organs, evidence of the involvement of these genes in oro-pharyngeal development is very limited. Niikawa et al. [1] reported the prevalence of CP in KS as 33% and Schrander-Stumpel et al. [10], reported that 50% of patients with KS had CP or bifid uvula. Although Lida et al. [11] reported six patients with KS with CP, few reports have discussed the characteristics of CP in KS and the outcomes of postoperative speech development. The purpose of this study is to investigate the characteristics and surgical outcomes of CP in patients with KS, and to discuss the importance of proper diagnosis of CP or SMCP and the determination of the surgical method.

## Methods

**Fig. 1:**
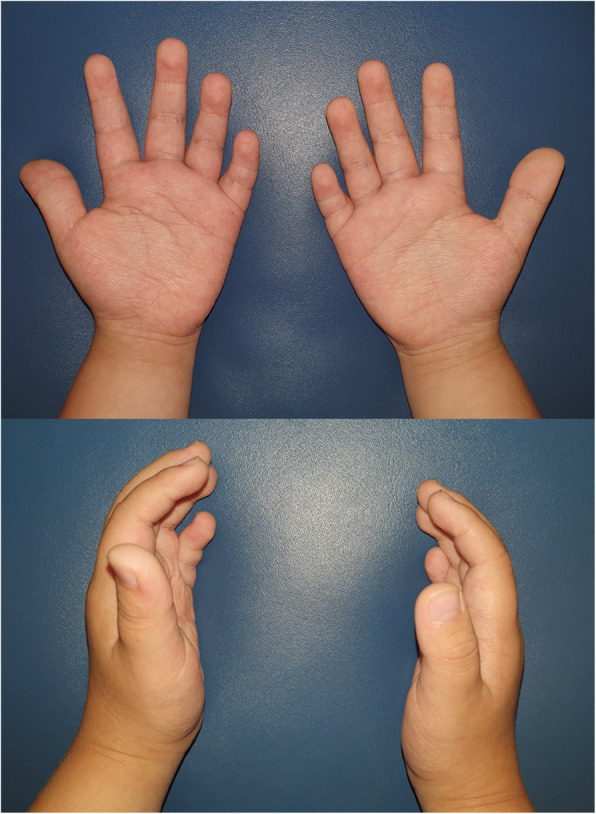
Characteristic clinical features of a patient with Kabuki syndrome: short fifth finger and persistence of fingerpads

This study was approved by the Institutional Review Board of the committee of Seoul National University Bundang Hospital (Number:B-2103/673–108). Informed consent to publish from legally authorized representative of the minor for Fig. 1 has been obtained. We conducted a retrospective study on patients with KS previously examined in the Department of Plastic and Reconstructive Surgery and the Department of Pediatrics of Seoul National University Bundang Hospital between 2003 and 2019. All patients were clinically diagnosed using diagnostic criteria [6] and genetically diagnosed patients were recorded. (Fig. 1) Patients who did not undergo surgery or who were lost to follow-up were excluded. The clinical data and surgical outcomes of 11 patients were collected from the patients’ medical records. Velopharyngeal function was evaluated using nasopharyngoscopy and speech analysis. Postoperative results were assessed by speech evaluation which was performed by the speech pathologist (Ahn) and the presence of velopharyngeal insufficiency (VPI), including remaining hypernasality (grade > 1 by Henningsson rating system) was evaluated. To investigate the correlation between postoperative speech outcomes and the presence of KS, statistical analysis using Fisher’s exact test was performed with patients who had previously operated non-syndromic CP and SCMP in our institute [12, 13]. A *p-value* ≤ 0.05 was considered statistically significant. All statistical analyses were performed using SPSS version 22.0 (SPSS Inc., Chicago, IL).

### Palatoplasty methods

Double opposing Z plasty (DOZ) and limited incision with thorough elevation (LITE) palatoplasty were used for correction of CP. The primary surgical procedure was performed when the patients were approximately 12–18 months of age. In cases of CP patients for whom preoperative evaluation was impossible, palatoplasty method was decided according to the surgical protocol of our institute. LITE was performed when the cleft gap was greater than 1 cm or the complete type, and DOZ was performed when the cleft gap was less than 1 cm. In cases of SMCP or VPI following primary palatoplasty, DOZ and posterior pharyngeal flap (PPF) were used for correction when the patient reached the age of cooperating with nasopharyngoscopy,. The surgical method was comprehensively determined based on the results of speech evaluation and nasopharyngoscopy.

DOZ was performed as described by Furlow with slight modifications [14]. Intraoperatively, all abnormally inserted levator veli palatine muscles were fully released and anatomical reconstruction was achieved. As per our modification, the extent of muscular dissection was greater than that described by Furlow, which released all abnormal insertions along the posterior border of the hard palate [12]. LITE is a modified two-flap palatoplasty that limits the incision at the anterior hard palate and elevates the movable palatal flap with thorough dissection over the entire palate [13]. DOZ or LITE was considered to be ineffective when the results of speech evaluation and nasopharyngoscopy showed that the palatal muscle was incompetent, and PPF was performed to create an anatomical barrier between the oral cavity and the nasal cavity to reduce nasal emission [15]. The PPF procedure involves elevation of a superior-based myomucosal flap and insertion into the soft palate to create a static sling and overcome the VPI.

### Speech evaluation

An experienced speech pathologist (Ahn) performed standardized speech evaluation for these patients. Perceptual speech evaluation was performed using the universal parameters and rating system described by Henningsson which consists of hypernasality, hyponasality, nasal emission, articulation errors, and intelligibility [16]. In addition to perceptual assessment, nasalance score, which is a ratio between oral and nasal acoustic energy, was obtained using Nasometer II 6400 (KAYPENTAX, Montvale, NJ). The nasometry was used as a supplementary tool in patients with hypernasality in speech evaluation, where it was used to aid in the interpretation of the results of hypernasality For all parameters, a score of 0 indicated that, within normal limits, there was no deviation from present perception.

### Nasopharyngoscopy

Nasopharyngoscopy was performed in VPI patients after the first palatoplasty and SMCP patients who were cooperative, usually > 4 years. A flexible fiberoptic endoscope was inserted for evaluation of velopharyngeal motility and visualization of the anatomy under various conditions and phonations. VPI was evaluated by closure of the velopharyngeal port during phonation [15]. To determine the surgical plan preoperatively, or to evaluate postoperative outcomes, the size of the central gap was measured with the lateral pharyngeal walls maximally contracted to the velopharyngeal port. The size of the central gap and the symmetry of lateral pharyngeal wall movement were recorded while the patients repetitively pronounced oral and nasal pressure-loaded words. The size of the central gap was classified into six categories. 0, closure; 1, touch closure (pinhole); 2, small (close ≥80% of the resting gap); 3, intermediate (close to 50–80% of the resting gap); 4, large (close to < 50% of the resting gap); and 5, hypodynamic velopharynx]. The lateral pharyngeal wall movement was categorized as either symmetric or asymmetric. The velopharyngeal closure pattern was classified into four categories as follows: 1, coronal; 2, circular; 3, sagittal; and 4, bow tie. If the postoperative speech outcome was within normal limits in follow-up period, no additional nasopharyngoscopy was performed.

## Results

**Fig. 2:**
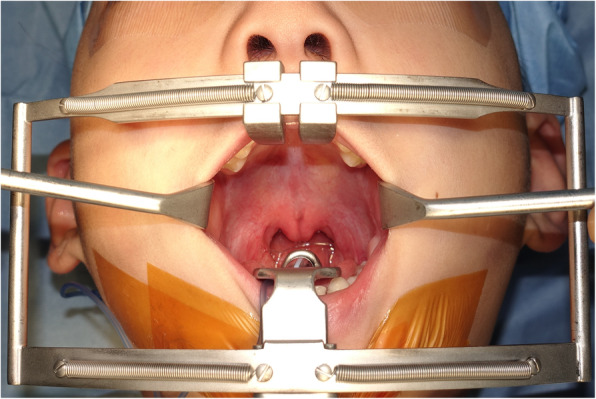
Intraoperative photo. Submucous cleft palate with bifid uvula and zona pellucida

Sex, type of CP, combined congenital anomalies, and clinical characteristics, including major diagnostic criteria are summarized in Table 1. The included patients comprised five men (45.5%) and six women (54.5%) and the mean follow-up period was 5 years 7 months (2 years to 11 years and 2 months). Four patients were genetically tested, and all were found to have pathogenic variants of *KMT2D*. For the other seven patients with KS, genetic tests were not performed due to parental refusal. Five patients had CP (45.5%) and six patients had SMCP (54.5%). All patients with CP were of incomplete type, and incomplete clefts of three patients (cases 1,2, and 5) involved hard and soft palates. The other two patients (cases 3 and 4) had clefts involving only the soft palate. All patients with SMCP had the classic triad of bifid uvula, hard palate bony notch, and zona pellucida of the soft palate (Fig. 2).

**Table 1:** Clinical characteristics of patients

	Sex	Type	Major diagnostic criteria					Genetic diagnosis	Other congenital anomalies
			(1)	(2)	(3)	(4)	(5)		
1	F	CP	O	O	O	O	O	–	VSD
2	M	CP	O	O	O	O	O	–	
3	F	CP	O	O	O	O	O	–	ASD / Blepharoptosis, Rt.
4	M	CP	O	O	O	O	O	–	ASD / Scoliosis
5	M	CP	O	O	X	O	O	O (KMT2D)	
6	M	SMCP	O	O	O	O	O	–	
7	F	SMCP	O	O	O	X	O	–	
8	F	SMCP	O	O	O	O	O	O (KMT2D)	Strabismus
9	F	SMCP	O	O	O	O	O	O (KMT2D)	Strabismus
10	F	SMCP	O	O	X	O	O	–	
11	M	SMCP	O	O	O	X	O	O (KMT2D)	Hypothyroidism, Horseshoe kidney

Major diagnostic criteria

(1) long palpebral fissures with eversion of the lateral third of the lower eyelid

(2) arched and broad eyebrows with the lateral third displaying notching or sparseness

(3) short columella with depressed nasal tip

(4) large, prominent or cupped ears

(5) finger fat pads

*CP* Cleft palate, *SMCP* Submucous cleft palate, *VSD* Ventricular septal defect, *ASD* Atrial septal defect)

The surgical methods, age at surgery, results of preoperative and postoperative speech evaluation, and nasopharyngoscopic findings are summarized in Table 2. Preoperative nasopharyngoscopic findings revealed an average central gap size of 3.1 points. Of the five patients with CP, three patients underwent LITE palatoplasty and two patients underwent DOZ palatoplasty. Four patients (cases 1,3,4 and 5) were unable to achieve velopharyngeal competency and had remaining hypernasality at postoperative follow-up. Among the four patients with SMCP who underwent DOZ palatoplasty, only one patient achieved velopharyngeal competency. Intraoperatively, muscular deficiency was identified in all patients who underwent palatoplasty. Two of the patients with SMCP underwent PPF as the first operation and five patients (three patients with CP and two patients with SMCP) underwent PPF as the second operation. All seven patients who underwent PPF operation achieved velopharyngeal competency. In previous studies conducted by our institute, in non-sydromic patients, 54 of 56 patients with CP (96.4%) and 54 of 64 patients with SMCP (84.3%) obtained velopharyngeal competency. Fisher’s exact test showed statistically significant difference in postoperative results between non-syndromic and KS patients. In patients with CP and SMCP, the *p*-values were < 0.001 and 0.02, respectively, and these results showed that surgical outcomes were poor in patients with KS (Table 3.)

**Table 2:** Palatoplasty, Speech evaluation and Nasopharyngoscopy results

	Sex	Type	Operation (Age at operation, in years and months)		Preoperative Nasopharyngoscopy	Speech evaluation		
			1st	2nd		Pre (1st)	Pre(2nd)	Post
1	F	CP	LITE (1y3m)	(−)	(−)	(−)	(−)	2/0/2/1/2
2	M	CP	LITE (1y6m)	(−)	(−)	(−)	(−)	1/0/2/1/1
3	F	CP	LITE (1y6m)	PPF (7y6m)	4/1/1	(−)	3/0/2/1/2	1/0/2/1/1
4	M	CP	DOZ (1y8m)	PPF (6y8m)	2/1/3	(−)	2/0/1/1/2	0/0/1/1/2
5	M	CP	DOZ (1y5m)	PPF (5y6m)	3/1/2	(−)	2/0/2/1/2	0/0/0/1/1
6	M	SMCP	DOZ (5y12m)	PPF (7y3m)	4/1/2	3/0/2/1/2	2/0/2/1/2	0/0/0/1/0
7	F	SMCP	DOZ (5y11m)	PPF (7y2m)	3/1/2	3/0/2/1/1	2/0/2/1/1	1/0/2/1/1
8	F	SMCP	DOZ (5y10m)	(−)	2/1/2	3/0/2/1/2	(−)	2/0/2/1/2
9	F	SMCP	DOZ (6y5m)	(−)	4/1/2	2/0/2/1/2	(−)	0/0/2/1/2
10	F	SMCP	PPF (6y1m)	(−)	3/1/2	2/0/2/1/2	(−)	0/0/0/1/0
11	M	SMCP	PPF (5y10m)	(−)	3/1/2	2/0/1/1/2	(−)	0/0/1/1/0

Speech evaluation: hypernasality / hyponasality / nasal emission / articulation errors / intelligibility

Preoperative nasopharyngoscopy: Opening size / Symmetry / Pattern (1, coronal; 2, circular; 3, sagittal; 4, bow tie)

*CP* Cleft palate, *SMCP* Submucous cleft palate, *LITE* Limited incision thorough elevation palatoplasty, *PPF* Posterior pharyngeal flap, *DOZ* Double opposing Z-plasty

**Table 3:** Statistical analysis (Fisher’s exact test performed by SPSS version 22.0)

Group	HN(−)	HN (+)	***p***-value
**CP**			< 0.001
Non-syndromic	54	2	
Kabuki	1	4	
**SMCP (DOZ)**			0.02
Non-syndromic	54	10	
Kabuki	1	3	

*HN* Hypernasality

## Discussion

### Characteristics of CP and diagnosis of submucous CP in KS

Handa et al. reviewed patients with KS with cleft lip, CP and SMCP, and reported that 41% of these patients had cleft lip and palate [17]. Niikawa et al. reported that 23 of 56 patients with KS have CP/lip including isolated CP, isolated cleft lip and CP with cleft lip [1]. However, Burke and Jones [18] and Schrander-Stumpel et al. [10] reported that all of patients with KS with CP were of the isolated CP type. In our study, all patients had isolated CP or SMCP without cleft lip or alveolar cleft. Clinical findings, such as gap size and hard palate involvement, were not significantly different from those of other non-syndromic patients with CP. Four genetically diagnosed patients had a heterozygous pathogenic variant of *KMT2D*, which was consistent with the results of previous studies showing CP was more associated with the *KMT2D* gene [6]. Previous studies reported that most patients with CP and KS have pathogenic variants in *KMT2D*, which may also be related to increase feeding problems, speech delays, and orodental defects [19]. To date, only one case series by Lida et al. examined the SMCP of patients with KS [11]. They reported that 6 patients had CP and 3 of 6 (50%) had SMCP. Moreover, they suggested that mental retardation, which is often accompanied by KS [20], is an obstacle for diagnosing SMCP because delayed speech development could be also attributed to be caused due to mental retardation. There have been no large studies on the proportion of SMCP in patients with KS. This study is the first report describing association of a pathogenic variant of *KMT2D* with SMCP in patients with KS. In this study, 6 of 11 patients (54.5%) had SMCP, all of whom were referred for delayed speech development. Among the six patients, five were not diagnosed with KS at birth. Three genetically diagnosed patients with SMCP had a heterozygous pathogenic variant of *KMT2D.* KS is difficult to diagnose in neonates and infants because their facial features are not as obvious as those of older children [3]. One of these patients (case 8), despite being diagnosed with KS in another hospital, was not suspected to have SMCP for delayed speech development. Based on these results, it is important to consider that there is a higher possibility of SMCP in KS than previously reported. Further studies will be needed on the incidence of SMCP in patients with KS with a pathogenic variant of *KMT2D*.

### Delayed speech development in KS

A considerable number of patients with KS show delayed speech development [9]. Mental retardation or cognitive delays are a common characteristic of patients with KS and these features are important causes of delayed speech development in patients with KS. Upton et al. [21] suggested that speech delay appeared to be due to poor coordination and oral-motor hypotonia, not structural abnormalities. However, as mentioned above, there is a possibility that an underdiagnosed SMCP in patients with KS is an obstacle to speech development. Therefore, the speech developmental delay in patients with KS appears to be due to various factors including mental retardation, oral-motor hypotonia, and undiagnosed SMCP [22, 23]. In our series, case 4 had mental retardation and it was difficult to evaluate a certain cause of delayed speech development. Although there is a limit to improving intelligibility or expression skills, speech problems resulting from structural abnormalities, such as hypernasality, can be corrected by proper operation. Therefore, to evaluate the speech development of patients with KS, it is essential to distinguish between a pure delay of speech development and a delay combined with SMCP through precise speech evaluation of hypernasality, nasal emission, articulation error and intelligibility [24]. If corrective surgery is appropriately performed, especially in patients with a normal range of intelligence or mild mental retardation, there can be considerable improvement in language development (cases 6 and 8).

### Preoperative evaluation and determination of surgical methods

**Fig. 3:**
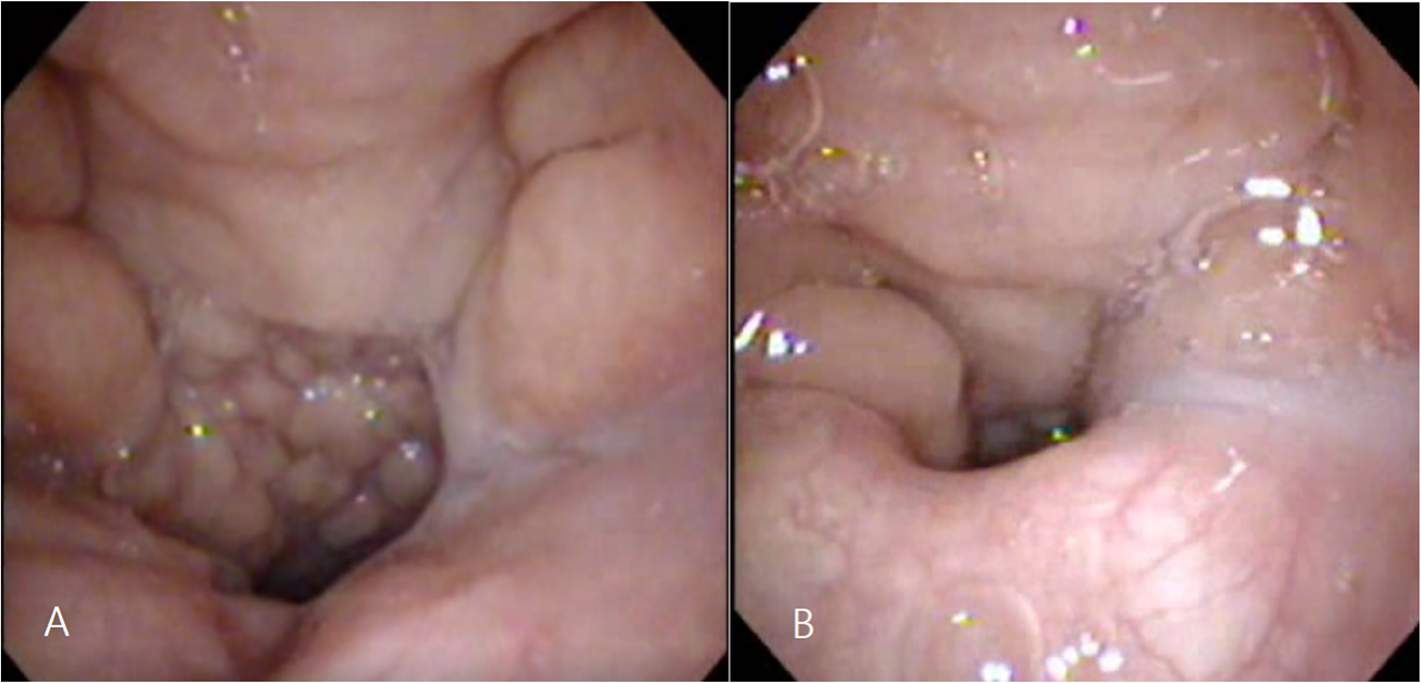
Measuring the size of central gap with nasopharyngoscopy. Velopharyngeal port on Resting state (left) and Maximally contracted state (right)

**Fig. 4:**
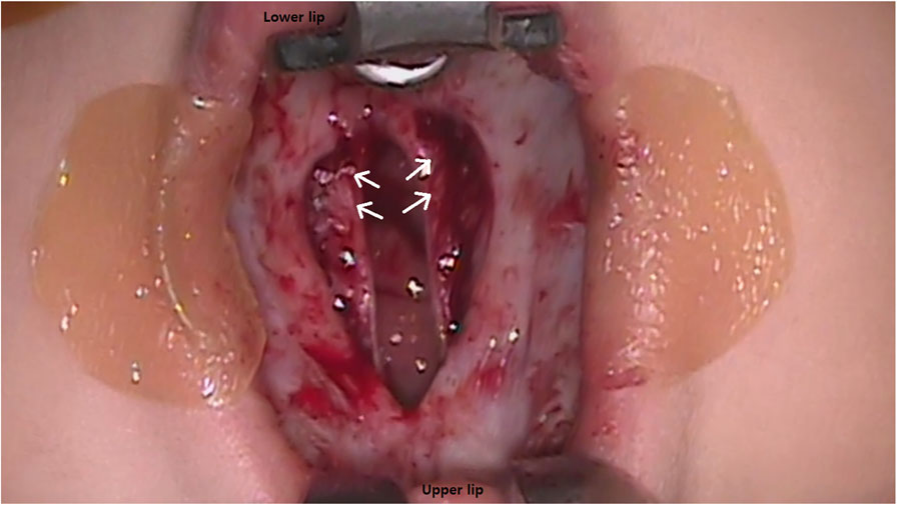
Intraoperative photo showing deficiency of the levator veli palatini muscle (white arrows)

In addition to speech evaluation, nasopharyngoscopy was performed on all patients with SCMP and CP who underwent a second surgery for velopharyngeal insufficiency correction. Other imaging studies, such as videofluoroscopy, also represent good tools for diagnosis [25, 26]. In preoperative planning, especially planning for PPF surgery, the size of the flap needs to be accurately measured and correctly designed to fill the central velopharyngeal gap. Although operator proficiency and patient cooperation are required, nasopharyngoscopy is the best tool for preoperative planning (Fig. 3). Compared to the results in our institute [12, 13], patients with CP or SMCP who underwent palatoplasty showed unsatisfactory results, such as persistent hypernasality, which was also statistically significant (Table 3). Based on these results, it is assumed that the postoperative results of patients with KS are not satisfactory compared to patients who had similar degrees of severity in preoperative findings. Therefore, as poor outcomes have been reported with conventional palatoplasty in other syndromic patients [27–29], it is also necessary to consider the possibility of unsatisfactory results due to factors separate from mental retardation in patients with KS. Antonio et al. reported that DOZ palatoplasty produced satisfactory results in non-syndromic patients but poor results in the velocardiofacial syndrome (VCFS) group; of the four reviewed patients, none had adequate velopharyngeal closure [28]. Chegar et al. [30] reported that pharyngeal flap surgery was the most effective treatment for patients with VCFS who had velopharyngeal insufficiency with hypernasal resonance. Case 11 had an intermediate opening size, for which the PPF was not considered when compared to previous conventional cases. However, the PPF surgery was performed based on speech evaluation, movement of the palate on nasopharyngoscopy and experience of the previous KS cases, and satisfactory results were achieved. Park et al. [31] reported that the thickness of the levator veli palatini muscle in patients with VCFS was significantly lower than that in non-syndromic patients with SMCP. We observed a similar deficiency of levator veli palatini muscle intraoperatively in patients with KS (Fig. 4). This could be a contributing factor to suboptimal results after surgical correction and further studies should follow. The surgeon must keep in mind that it is difficult to produce optimal results with conventional palatoplasty in the absence of muscular structure, and this should be clearly explained to the parents of the patients. All seven patients who underwent pharyngeal flap surgery (cases 3–7, 10, and 11) showed satisfactory results. Therefore, although palatoplasty is performed to close the nasal and oral cavity in cases of congenital CP, it is important to be aware that there is a high possibility of velopharyngeal insufficiency and pharyngeal flap surgery should be considered as proper treatment in patients with KS.

## Conclusion

The results of this study suggest that there are more cases of SMCP in patients with KS than previously reported, and SMCP should be considered when this patient group present with speech problems. We also observed deficiency of the levator veli palatini muscle, which made it difficult to produce optimal results with conventional palatoplasty in patients with KS. Therefore, pharyngeal flap surgery should be considered as a proper treatment to obtain favorable results and this method should be carefully designed based on speech evaluation and nasopharyngoscopic findings.

### Supplementary Information

**Additional file 1: Supplementary material 1.** Nasopharyngoscopic finding. A flexible fiberoptic endoscope was inserted for evaluation of velopharyngeal motility and visualization of the anatomy under various conditions and phonations. This patient with kabuki syndrome showed velopharyngeal insufficiency associated with hypernasality and intermediate size of central gap.

## Data Availability

Data has been anonymized and is kept with the authors. Datasets are available from the corresponding author on reasonable request.

## Abbreviations

KS: Kabuki syndrome
CP: cleft palate
SMCP: Submucous cleft palate
DOZ: Double opposing Z plasty
LITE: Limited incision with thorough elevation
VPI: Velopharyngeal insufficiency
PPF: Posterior pharyngeal flap
VCFS: Velocardiofacial syndrome
